# Medical News

**Published:** 1852-03

**Authors:** 


### MEDICAL NEWS.

PENNSYLVANIA HOSPITAL.—The Board of Managers of this Institution have resolved to appoint an additional attending surgeon, which increase will make the number of surgeons *four* instead of *three*, as at present. The election for this post will be held on the first Monday in May.

MEDICAL DEPARTMENT OF THE ARMY.—By a Board of Army Surgeons, which recently convened in the city of New York for the examination of Assistant Surgeons for promotion, and of candidates for appointment in the Medical Staff of the Army, the following named gentlemen were examined and approved.

*Assistant Surgeons.*—Levi H. Holden, Richard F. Simpson, Robert Murray, and Lewis A. Edwards.

*Candidates for Appointment.*—Basil Norris, Md.; T. Charlton Henry, Pa.; Andrew J. Foard, Ga.; Edward P. Vollum, N. Y.; John Moore, Ind.; Andrew K. Smith, Conn.; Edward H. Watson, Pa.; Richard Potts, Md.; Richard H. Alexander, Ky.; and George Suckley, N. Y.

A Board of Naval Surgeons convened on the 16th ult., at the U. S. Naval Asylum, for the examination of Assistant Surgeons for promotion. The Board consisted of Surgeon Thomas Dillard, President, and Drs. Horner, Mosely, Blacknall, and Hunter, members.

Dr. Valentine Mott has been recalled to the Professorship of Surgery, and the Presidency of the Medical Department of the University of New York. It is not known whether the Doctor will accept. The effort having failed to secure Dr. Goddard, of Philadelphia, for the chair of Anatomy, vacated by the death of Dr. Pattison, it is thought that Dr. Van Buren will be appointed to that post.

Extramural Interments in London.—Lord J. Russell has lately announced, that the Government of Great Britain would shortly introduce a bill to facilitate the promotion of this desirable hygienic reform.

The Mummy found in St. Stephen's Crypt, Westminster.—On Saturday, 31st January last, a minute examination of the mummy found a few days previously, took place in London. The body, after being unrolled, was removed from its narrow cell and placed upon a bench. An incision having been made down the centre of the abdomen, and in a lateral direction round the skull, a layer of fine thick canvass cloths was removed from off the face. A second series bound round by string having then presented themselves, they were in due course loosened, and the face was disclosed in a remarkable state of preservation. The cartilage of the nose was not at all decayed, and, with the lips and other portions of the face, remained perfectly flexible to the touch. Even the expression of the countenance was in a degree retained, and it was remarked that the identity of the individual would not have been impossible, had any compeer of his venerable age been present. The stomach was found to have retreated from the cloth, and to have become a mass of adipose matter, in which state the legs and arms were also found. No writing of any description was discovered in the folds, nor was any mark leading to an identity of the individual found. The body measured 5 feet 11 inches in length; and, judging from the front teeth remaining, three or four of which in the lower jaw were much worn, must have been that of a very aged man. The mouth was filled with tow, which had evidently been steeped in wax; and a small quantity of hair remained on the chin and upper lip. The crozier was entirely of oak, with an elaborately carved crook—the whole measuring 6 feet 2 inches in length. The gentlemen present at the examination unanimously agreed, that the presumption of its being Lydwolfe, Bishop of St. David's, who died about the middle of the fifteenth century, was almost indisputable.

Death of M. Gannal.—The Paris obituary list of the last week in January contains a name of some celebrity, that of M. Gannal, the inventor of the new embalming system. His career was a singular one. Apprenticed to an apothecary in early life, he imbibed that taste for, and acquired that knowledge of chemistry, which subsequently proved so serviceable to him. At the commencement of the century, the conscription forcibly took him from his favorite studies. In a short time, he became attached to the medical corps of the French army in Germany, and was present at some of the great battles of Napoleon against Prussia and Austria, and formed part of the medical staff in the Russian campaign. In the disastrous retreat which followed, he was taken prisoner at Wilna, but on four occasions succeeded in making his escape, and was as often recaptured. After a thousand adventures by flood and field, in 1815 he returned to France, where his acquirements soon obtained for him a place in the School of Pharmacy, and he made several curious discoveries in chemistry, which, however, with the exception of a prize at the Academy of Sciences, procured him no real advantages; until his great discovery of embalming by means of a chemical preparation, which in a few years made him master of a large fortune. M. Gannal's account of his process was published in 1839, in this city, translated by the late Dr. Richard Harlan.

Naval Assistant Surgeons.—The cause of the Naval Assistant Surgeons progresses. It will no doubt be a subject of congratulation to to the gentlemen in the Service to know, that Admiral Houston Stewart, one of the Lords of the Admiralty, and Mr. Montague Chambers, Q.C, both being candidates to represent the borough of Greenwich in Parliament, have promised to insist that the Admiralty Order of July, 1850, shall be carried out in strict integrity and without evasion. We hoped to publish the correspondence that has taken place on this subject between the respective candidates and Mr. T. M. Stone; but, for certain reasons the naval candidate objects to the publication of his letters.

Mr. Chambers, however, states, that the subject to which his attention had been drawn by Mr. Stone, had occupied his thoughts long before he was solicited to become a candidate to represent Greenwich; that it was his opinion the Naval Assistant-Surgeons should occupy a very different position as men of education, competent to discharge such serious and essential duties so intimately connected with the welfare, the safety, and the comfort of the Naval service. "I assure you most sincerely," says Mr. Chambers, "that I am thoroughly convinced of the necessity of the Admiralty Order, and that to the utmost of my power I will afford any assistance I can, as a Member of Parliament or otherwise, in causing it to be carried into execution according to *its true spirit and object;*" and, in conclusion, Mr. Chambers states, that "the condition of the other medical officers must be improved, and placed on a similar footing to their more fortunate brethren in the Army." Fearing he had not been sufficiently explicit, he again wrote to Mr. Stone, expressing his regret that he was not at home when that gentleman called on him, as it would have enabled him to confer with Mr. Stone "upon the most useful way of serving the cause of the Naval Assistant-Surgeons, and glad to have the opportunity of expressing my sentiments, and affording even the most trifling aid in so good a cause."

It is generally understood in Greenwich, that both the gentlemen will be returned to parliament. This will then be a great addition to the ranks of those members who have already so nobly supported Captain Boldero in his efforts to improve the position of the Naval Assistant-Surgeon.—*Lond. Med. Times.*

### MR. SYME'S ORIGINALITY.

*To the Editor of the Medical Times.* Sir,—I read in your replies to correspondents a notice regarding my former master, Professor Syme. His wonderful operations have, as is well known in Edinburgh, excited the jealousy of London surgeons, who are still unwilling to admit that all discoveries in surgical science for the last forty years have emanated from our University. Who, before Mr. Syme, introduced the excision of joints? Who, before Mr. Syme, operated at the ankle-joint? As to your notice of the perineal section, stated to have been performed by a French surgeon of little note, I ask, where are the cases which he has recorded? and why are we to believe your simple assertion in preference to others? As for the lip operation, so peculiarly his own, who ever dreamed of restoring that important organ before Mr. Syme? In diagnosis, too, Mr. Syme is notoriously pre-eminent. Who but he detected an aneurism of the carotid, and tied it too, when his colleague had diagnosed a strumous gland? Who but he cut down upon the iliac, when others only saw a malignant tumor of the groin? You may attack the professional characters of those whom you and your compeers can never hope to equal: but I tell you that there are plenty who, with myself, are willing to believe in Mr. Syme's statement, before all the medical journals put together, and ready to take up more than the cudgel in his defence. I am, &c.

Edinburgh. A Former Pupil of Mr. Syme.

[As regards our correspondent, it is evidently not true that "the ass knoweth his master's *crib.*"—Ed. *Med. Times.*]

A BRAZEN HOMŒOPATH.

To the President of the Royal College of Physicians.

Sir,—I am anxious to know if you will admit me to an examination for the degree of Doctor of Medicine, and as I wish to act honestly and fairly to you, I think it best to state, that I differ from the views of the College in one branch of the examination—viz., the therapeutical. However, I will give my answer to any question you put according to the old mode of healing disease, and at the same time, if agreeable to you, state what homœopathists consider the best method of curing disease. I trust the fact of my being a homœopathist, which I am from conviction and long experience, will not prevent you from granting me that diploma which I am desirous of obtaining. I have the honor to be, &c., &c.

*Reply.*

Sir,—The foundation of the Royal College of Physicians was for the purpose of guaranteeing to the public skilful and safe practitioners. The College of Physicians regard the so-called homœopathists as neither skilful nor safe practitioners. Therefore the College cannot, without betraying a sacred trust, give its license to persons whom they regard as wholly unworthy their confidence, and with whom it is not possible they can hold any communion. I remain, sir, your obedient servant,

*Dublin Med. Press.*] J. A. Paris.

Medical Charities—The following princely donations have been bequeathed to the undermentioned medical institutions by the late Mr. Thomas Dickinson, of Upper Holloway. The Idiot Asylum, 2000*l.*; the London Hospital, 1000*l.*; the Royal Free Hospital, 1000*l.*; the Charing Cross Hospital, 1000*l.*; the Fistula Infirmary, 1000*l.*; and the Holloway Dispensary, 500*l.* In addition to the above munificent sums to medical charities, this genteman has left similar large amounts to various non-medical institutions.—*Lond. Med. Times.*

HONORS OF MEDICAL MEN.—It has pleased the Queen to grant permission to Mr. Owen, the distinguished professor of Comparative Anatomy at the College of Surgeons, to reside in one of the houses on Kew Green which belonged to the late King of Hanover. The gift was accompanied by a very flattering letter from Prince Albert to the professor. Another of the houses on the same green has, we understand, in a like kindly spirit, been presented for a residence during life to Dr. Joseph Hooker. We have also to record a mark of royal favor bestowed by a foreign sovereign on Professor Owen. Our readers know that the King of Prussia instituted some time since an Order of Merit, for men distinguished in art and in science—and which consists of sixty chevaliers, thirty Prussians and thirty foreigners. Amongst the latter was the late Professor Oersted; and Professor Owen has been selected by Frederick William to supply the vacancy occasioned by his death. In this order there are now five Englishmen:—Mr. R. Brown, Sir David Brewster, Sir J. Herschel, Mr. Faraday and Professor Owen.—*London Lancet.*

MUNIFICENT DONATION.—The Countess de la Riboisière has left all her property to the city of Paris, the surviving Count retaining a life-interest. This bequest, which is said to amount to the enormous sum of £320,000, is intended for the foundation of an hospital, which is to bear the name of La Riboisière.—*Ibid.*

BARON PASQUIER, formerly surgeon to Louis Philippe, died at Paris on the 4th of January, 1852. His widow has been granted a pension from Government, as she was left without property.—*Ibid.*

MEDICAL MUSEUMS.—The famous toxicologist, Professor Orfila, ex-dean of the Medical Faculty of Paris, has recently returned from visiting the chief medical museums of Germany. He had previously explored those of Italy, Spain, and England. The palm of superiority he awards to France; though he admits that in some things the French museums are excelled by those of London and other places. In comparative anatomy, the museum of our College of Surgeons, and the Berlin museum of normal anatomy, take the foremost rank. The collection of skeletons contained in the latter is unrivalled, both for the number and beauty of the preparations. *L'Union Médicale*, of 11th December, devotes a *feuilleton* to Orfila's notes on the German museums.—*London Journ. of Medicine.*

International Sanitary Convention in Paris.—It was certainly a happy idea to collect in Paris, delegates from all the countries bathed by the Mediterranean, in order to frame laws and regulations, which should place the sanitary relations of the various countries upon an uniform basis. This convention, composed principally of medical men, consuls, &c., has just closed its meetings, which took place once a week regularly for the last six months; and plans for international sanitary relations have been framed and placed in the hands of the French Goernment. When these plans have been ratified by the respective powers, they will be published and put in force. It is, however, already known that the system of quarantine will be thoroughly changed; in fact, orders have already been given by the French Government to pull down the lazaretto at Marseilles, and convert it into docks. We shall just mention, *en passant*, that each member of the convention has been courteously decorated with the cross of the Legion of Honor. When shall we have marks of distinction bestowed upon deserving medical men in this country?—*London Lancet.*

Arsenic Eaters.—A trial for murder took place lately in Austria, in which the prisoner, Anna Alexander, was acquitted by the jury, who, in the questions put to the witnesses, in order to ascertain whether the murdered man, Lieutenant Mathew Wurzel, was a poison-eater or not, educed some curious evidence relating to this class of persons. As it is not generally known that eating poison is actually practised in more countries than one, the following account of the custom, given by a physician, Dr. T. von Tschudi, will not be without interest. In some districts of Lower Austria and in Styria, especially in those mountainous parts bordering on Hungary, there prevails the habit of eating arsenic. The peasantry in particular are given to it. They obtain it under the name of *hedri* from the travelling hucksters and gatherers of herbs, who, on their side, get it from the glass-blowers, or purchase it from the cow-doctors, quacks, or mountebanks. The poison-eaters have a two-fold aim in their dangerous enjoyment; one to obtain a fresh, healthy appearance, and acquire a certain degree of *embonpoint.* The number of deaths in consequence of the immoderate enjoyment of arsenic, is not inconsiderable, especially among the young. Every priest who has the cure of souls in those districts where the abuse prevails, could tell of such tragedies; and the inquiries made by Dr. Tschudi on the subject have opened out very singular details. The second object the poison-eaters have in view, is to make them, as they express it, "better-winded!"

—that is, to make their respiration easier when ascending the mountains. Whenever they have far to go, and to mount a considerable height, they take a minute morsel of arsenic, and allow it gradually to dissolve. The effect is surprising; and they ascend with ease heights which otherwise they could climb only with effort. The dose of arsenic with which the poison-eaters begin, consists, according to the confession of some of them, of a piece of the size of a lentil, which in weight would be rather less than half a grain. To this quantity, which they take fasting several mornings in the week, they confine themselves for a considerable time; and then gradually, and very carefully, increase the dose according to the effect produced. A strong, hale man, upwards of sixty, takes at present at a dose a piece of about the weight of four grains. For more than forty years he has practised this habit, which he inherited from his father, and which he in his turn will bequeath to his children. It is well to observe, that neither in these nor in other poison-eaters is there the least trace of an arsenic cachexy discernible; that the symptoms of a chronic arsenical poisoning never show themselves in individuals who adapt the dose to their constitution, even although that dose should be considerable. It is not less worthy of remark, however, that when, either from inability to obtain the acid, or from any other cause, the perilous indulgence is stopped, symptoms of illness are sure to appear, which have the closest resemblance to those produced by poisoning from arsenic. These symptoms consist principally in a feeling of general discomfort, attended by a perfect indifference to all surrounding persons and things, great personal anxiety, and various distressing sensations arising from the digestive organs, want of appetite, a constant feeling of the stomach being overloaded at early morning, an unusual degree of salivation, a burning from the pylorus to the throat, a cramp-like movement in the pharynx, pains in the stomach, and especially difficulty of breathing. For all these symptoms there is but one remedy—a return to the enjoyment of arsenic. According to inquiries made on the subject, it would seem that the habit of eating poison among the inhabitants of Lower Austria has not grown into a passion, as is the case with the opium-eaters in the East, the chewers of the betel-nut in India and Polynesia, and of the cocoa-tree among the natives of Peru. When once commenced, however, it becomes a necessity. In some districts sublimate of quicksilver is used in the same way. One case in particular is mentioned by Dr. von Tschudi, a case authenticated by the English ambassador at Constantinople, of a great opium-eater at Brussa, who daily consumed the enormous quantity of forty grains of corrosive sublimate with his opium.

In the mountainous parts of Peru the doctor met very frequently with eaters of corrosive sublimate; and in Bolivia the practice is still more frequent, where this poison is openly sold in the market to the Indians. In Vienna the use of arsenic is of every-day occurrence among horse-dealers, and especially with the coachmen of the nobility. They either shake it in a pulverized state among the corn, or they tie a bit the size of a pea in a peace of linen, which they fasten to the curb when the horse is harnessed, and the saliva of the animal soon dissolves it. The sleek, round, shining appearance of the carriage-horses, and especially the much admired foaming at the mouth, is the result of this arsenic-feeding. It is a common practice with the farm-servants in the mountainous parts, to strew a pinch of arsenic on the last feed of hay before going up a steep road. This is done for years without the least unfavorable result; but should the horse fall into the hands of another owner who withholds the arsenic, he loses flesh immediately, is no longer lively, and even with the best feeding there is no possibility of restoring him to his former sleek appearance. [The preceding extraordinary statement is abbreviated from a recent number of *Chambers's Edinburgh Journal,* in which it is given as authentic. The use of arsenic in improving the condition of horses is not unfrequent in this country, and its value in the cutaneous diseases of man is established. Such facts, in some degree, confirm these statements; but we entertain grave doubts that an agent of such uniform power as arsenic, could be commenced and habitually continued, in the large doses here mentioned, without producing effects the converse of those described.]—*London Lancet.*

